# Kin Recognition in the Parasitic Plant *Triphysaria versicolor* Is Mediated Through Root Exudates

**DOI:** 10.3389/fpls.2020.560682

**Published:** 2020-10-06

**Authors:** Yaxin Wang, Maylin Murdock, Seigmund Wai Tsuen Lai, Daniel B. Steele, John I. Yoder

**Affiliations:** Department of Plant Sciences, University of California, Davis, Davis, CA, United States

**Keywords:** Triphysaria, kin recognition, root exudate, 2,6-dimethoxy benzoquinone, parasitic plant, peroxidase, laccase, plant-plant interaction

## Abstract

*Triphysaria* is a facultative parasitic plant in the Orobanchaceae that parasitizes the roots of a wide range of host plants including *Arabidopsis*, *Medicago*, rice and maize. The important exception to this broad host range is that *Triphysaria* rarely parasitize other *Triphysaria*. We explored self and kin recognition in *Triphysaria versicolor* and showed that exudates collected from roots of host species, *Arabidopsis thaliana* and *Medicago truncatula*, induced haustorium development when applied to the roots of *Triphysaria* seedlings *in vitro* while those collected from *Triphysaria* did not. In mixed exudate experiments, *Triphysaria* exudates did not inhibit the haustorium-inducing activity of those from host roots. Interestingly, when roots of *Triphysari*a seedlings were treated with either horseradish peroxidase or fungal laccase, the extracts showed haustorium-inducing factor (HIF) activity, suggesting that *Triphysaria* roots contain the proper substrates for producing HIFs. Transgenic Triphysaria roots overexpressing a fungal laccase gene *TvLCC1* showed an increased responsiveness to a known HIF, 2,6-dimethoxy benzoquinone (DMBQ), in developing haustoria. Our results indicate kin recognition in *Triphysaria* is associated with the lack of active HIFs in root exudates. Treatment of *Triphysaria* roots with enzymatic oxidases activates or releases molecules that are HIFs. This study shows that exogenously applied oxidases can activate HIFs in *Triphysaria* roots that had no previous HIF activity. Further studies are necessary to determine if differential oxidase activities in host and parasite roots account for the kin recognition in haustorium development.

## Introduction

Kin- and self-recognition is a common theme in biology that occurs in bacteria, plants and animals (Keenan et al., 2001; Dudley and File, 2007). Animals recognize kin as an important factor in social behaviors as well as at the molecular levels: the animal immune system is highly sophisticated and regulates self-recognition system (Keenan et al., 2001; Chaplin, 2010). Plants are also capable of recognizing self or closely related plants at multiple levels. Sexual-incompatibility occurs widely in flowering plants to prevent fertilization by self or genetically related pollens which promotes out-crossing (Disorders et al., 2000; Takayama and Isogai, 2005; Zhao et al., 2010). Root growth observations on the desert shrub *Ambrosia* revealed that it is able to recognize and reduce its growth rate when growing with other *Ambrosia* roots (Mahall and Callaway, 1991; Mahall and Callaway, 1992). *Deschampsia caespitosa* are capable of detecting root exudates from plants of different genetic identities and change root morphology rather than root biomass in response to conspecific root exudates (Semchenko et al., 2014).

Parasitic angiosperms directly invade tissues of host plants to steal nutrients and water. The Orobanchaceae are a family of root parasitic plants that includes several agriculturally destructive species such as *Striga* and *Orobanche*. The range of suitable host plants varies widely among Orobanchaceae species. Most Orobanchaceae are generalists that can recognize and attach to a large number of co-occurring species (Shen et al., 2006). For example, *Triphysaria* and *Castilleja* parasitize over 100 hosts from a wide range of families including monocots and eudicots (Estabrook and Yoder, 1998; Jamison and Yoder, 2001; Press and Phoenix, 2005). Some parasitic plants are specialists and only parasitize a few hosts. For examples, *Striga gesnerioides* has an extensive host range of plants specifically in eudicots while *Striga hermonthica* parasitizes a number of monocots (Musselman, 1980). *Epifagus virginian* represents an extreme case of host specificity in Orobanchaceae by parasitizing only *Fagus grandifolia* (Press and Phoenix, 2005; Shen et al., 2006).

There are several stages in the progression of root parasitism at which a host is distinguished from a non-host, including germination stimulation, haustorium initiation, haustorium penetration, and post attachment physiological processes. We have focused on haustorium initiation and development because these stages are critical for the adaptation of parasitism in plants. The haustorium is the multifunctional organ that attaches the parasite to the host, invades the host and provides the physiological conduit for water and nutrients (Westwood et al., 2010). Facultative root parasites, such as *Triphysaria and Phtheirospermum*, develop lateral haustoria on the sides of roots, primarily in the elongation zone of root tips (Nickrent et al., 1979; Goldwasser et al., 2002; Cui et al., 2016). The development of haustoria in *Triphysaria* can be induced and monitored under a dissecting microscope either by adding a chemical haustorium-inducing factor (HIF) or by touching a host root to a *Triphysaria* root. Haustoria that develop without attaching to a host appear on parasite roots as hemispherical or spherical swellings covered by a localized proliferation of hairs. After host attachment, the haustoria are observed as root outgrowths connecting the host and the parasite (Jamison and Yoder, 2001).

In many Orobanchaceae, haustoria only develop in the presence of host roots whose exudates contain chemical HIFs (Yoshida et al., 2016). Although DMBQ is the only HIF isolated from host plants, a broad range of phenolics and quinones have been identified as active HIFs through *in vitro* applications (Smith et al., 1996; Albrecht et al., 1999; Cui et al., 2018). Some of these HIFs such as syringic acid, peonidine, xenognosin A&B are natural compounds present in plant extracts or root exudates (Lynn et al., 1981; Chang and Lynn, 1986; Strehmel et al., 2014). The current model of haustorium development in response to HIFs is that redox cycling between quinone and phenol states generates semiquinone intermediates that induce a redox sensitive signaling pathway (Smith et al., 1996; Matvienko et al., 2001; Bandaranayake et al., 2010).

Many parasitic Orobanchaceae including *Triphysaria*, *Striga*, *Agalinis* and *Phtheirospermum* only infrequently develop auto-haustoria in the absence of host roots, while other parasites, notably *Pedicularis*, develop spontaneous auto-haustoria abundantly (Steffens et al., 1982; Yoder, 1997; Cui et al., 2018; Xiang et al., 2018). Evolution of kin recognition was presumably an early event in plant parasitism, as parasitizing one’s own roots or those of nearby siblings having the same nutrients are unlikely to be fruitful (Westwood et al., 2010). The phenomenon of self and kin recognition associated with the lack of haustorium development in the parasitic plant *Triphysaria versicolor* has been previously recorded in Yoder, 1997. The aim of this study is to explore the mechanism of kin recognition in *Triphysaria*. Understanding how *Triphysaria* avoids being parasitized should suggest strategies for engineering resistance against parasitic weeds into crop plants. We analyzed exudates from host and parasite roots with and without enzymatic oxidation and showed that enzymatic oxidation eliminates kin recognition in *Triphysaria* seedlings. Our results suggest that the mechanism of *Triphysaria* kin recognition may be associated with the restricted release of HIFs in their root exudates, which can be activated by peroxidases and laccases.

## Materials and Methods

### Plants

Seeds of *Triphysaria versicolor*, *Triphysaria eriantha*, and *Triphysaria pusilla* were collected from different open pollinated populations in California. *Triphysaria versicolor* were growing in a pastureland south of Napa (GPS location: 38°13′33.2′′N, 122°16′11.7′′W). *Triphysaria eriantha* were growing in Jepson Prairie Preserve (GPS location: 38°16′33.3′′N, 121°49′21.8′′W). *Triphysaria pusilla* were growing in Bodega Bay Marine Reserve of UC Davis (GPS location: 38°19′′01.4′′N, 123°04′16.4′′W). *Arabidopsis thaliana* Columbia seeds were obtained from *Arabidopsis* Biological Resource Center. Seeds of *Medicago truncatula* (ecotype A17) were provided by Dr. Douglas R. Cook (University of California, Davis).

### Reagents

Horseradish peroxidase (516531, Millipore-Sigma, USA), laccase from *Trametes versicolor* (38429, Sigma-Aldrich, USA), 2,6-Dimethoxy-1,4-benzoquinone (DMBQ, 428566, Millipore Sigma, USA) were purchased from the indicated vendors.

### Haustorium Induction Assay

1. **Assay of haustorium development in *Triphysaria* seedlings treated with aqueous HIFs.** Haustorium development in *Triphysaria* was monitored following exposure to exudates, DMBQ and other HIFs (Jamison and Yoder, 2001). Approximately 1 week after germination, *Triphysaria* seedlings were aseptically transferred to square petri plates containing 0.25× Hoagland nutrient media (Hoagland and Arnon, 1950), 0.75% (w/v) sucrose and 0.75% (w/v) Phytagel (Sigma, USA). Twenty to fifty *Triphysaria* seedlings were transferred onto each plate. The square petri plates were placed vertically in 22°C growth chamber under a 16 h light regimen for 3 days prior to induction. Haustoria induction of *Triphysaria* was achieved by adding 1 ml of assay solution directly on top of root tips. Plates were kept horizontally for 2 h to allow absorption of the liquid inducer into the media and induce the tactile response of *Triphysaria* roots. The proportion of plants forming haustoria was counted 24 h later using a dissecting microscope.
2. **Assay of haustorium development in transgenic *Triphysaria* roots**. Transgenic *Triphysaria* roots were obtained by *Rhizobium rhizogenes* mediated transformation (Bandaranayake and Yoder, 2018). To assay haustorium development, 1 ml of 1 µM DMBQ was applied to the transgenic root tips and the proportion of roots that formed haustoria 24 h after treatment was counted.

### Root Exudates Collection

1. **Root exudates from *in vitro* root cultures**. We collected exudates from *in vitro* grown *Triphysaria* and *Medicago* root cultures (modified from Tomilov et al., 2004) and from *Arabidopsis* root cultures (modified from Czako et al., 1993). Aseptic *Triphysaria* or *Medicago* seeds were germinated on solid 0.25×Hoagland media and grown for 1 week before being placed into liquid 1× Hoagland medium supplemented with 2% (w/v) sucrose and 2 mg/L IAA. Aseptic *Arabidopsis* seeds were similarly germinated on solid 0.5× MS medium (Murasnige and Skoog, 1962), grown for 1 week and inoculated into liquid 1× MS medium supplemented with 2% (w/v) sucrose and 0.1 mg/L IAA. In all cases, the root cultures were kept dark in 500 ml cell culture flasks and shaken at 40 rpm in 22°C growth chamber. After 1 month the roots were cut from the seedlings and transferred into fresh 1× Hoagland with 2 mg/L IAA (*Triphysaria* and *Medicago*) or 1× MS media with 0.1 mg/L IAA (*Arabidopsis*) for 3 days after which the root cultures were transferred to fresh media without hormones. The three-day treatment with IAA was repeated every month for up to six months.
  Root culture media were collected from 2- to 6-month-old root cultures prior to the IAA treatment and concentrated using C18 Solid Phase Extraction (SPE) cartridges (Mega BE-C18, 5 gm 20 ml, Agilent Technologies, USA), which also removed sugar and minerals. SPE cartridges were conditioned with 20 ml 100% methanol and equilibrated with 20 ml H_2_O before loading samples. About 1L culture media were applied to the SPE cartridge by pulling a vacuum from the bottom of the cartridge to enhance liquid flow and controlling flow speed at about 3 ml/min. SPE columns were then washed with 30 ml 10% methanol and finally eluted in 30 ml 50% methanol. At least 3 biological replicates of root exudates were collected for each species. Root exudates were normalized based on total phenolic contents measured by Folin-Denis assay (Scalbert et al., 1989). Standard curve for total phenolic measurement was built using p-coumaric acid. The total phenolic concentrations of concentrated root exudates collected at different time or from different species varied from 60 µM to 220 µM. We mostly used root exudates at 100 µM total phenolic content, which is approximately 15 to 55 times concentration of the root exudates from the original culture media.
2. **Root extracts from enzymatically treated roots.** Roots of 2-week-old *Arabidopsis* or *Triphysaria* seedlings were harvested by cutting at the shoot-root junctions and rinsing gently with deionized water three times to remove cell lysate at the cutting site. Roots of twenty seedlings were incubated in 1 ml solution containing 14 mM MES buffer (PH 6), horseradish peroxidase (25 unit/ml) and H_2_O_2_ (0.005% v/v) or fungal laccase (25 unit/ml) with gentle shaking at room temperature for 15 minutes. Incubation of roots in 14 mM MES buffer without enzymes was used as control. Activity of purchased peroxidase (EC 1.11.1.7) and laccase (EC 1.10.3.2) were measured with syringaldazine as substrate following protocols by Sigma (Sheikhi and Roayaei, 2012). The enzymatic extraction solution was collected and filtered through Amicon Ultra-4 Centrifugal Filters (Pore size: 30 kDa, MilliporeSigma, USA) to remove the enzymes before assaying.

### DMBQ Quantification by LC-Triple Quadrupole MS

Quantification of DMBQ was carried out at the Food Safety and Measurement Facility at UC Davis (https://foodanalysis.ucdavis.edu). One ml of root exudates (at 100µM total phenolic contents) or enzymatic root extractions were concentrated by evaporating to dryness in a SpeedVac and resuspending in 20 to 50 µl 10% methanol to effectively concentrate the samples 20 to 50 times. Concentrated samples were analyzed using an Agilent 1100 LC system coupled to an Agilent 6460 triple-quadrupole MS system (Agilent Technologies Inc, USA) with positive electrospray ionization. Two µL injected samples were separated using a C18 column (ZORBAX Extend 80Å C18, 2.1 mm × 100 mm, 1.8 µm, Agilent Technologies Inc, USA) with binary mobile phase of A: water with 0.1% formic acid and B: methanol with 0.1% formic acid for a run time of 11 minutes. Data was acquired using multiple-reaction monitoring mode with protonated molecular ions (M+H)^+^ as precursor ions. Select settings were as follows: source temperature at 350°C, dwell time at 150 ms, ion spray voltage at 5500 V and curtain gas, gas 1, gas 2, and collision induced dissociation gases were respectively 50, 50, 60, and 4 psi. Internal standard calibration curves were built in each assay using pure molecule 2,6-dimethoxy-1,4-benzoquione (DMBQ, 428566, Millipore Sigma, USA) in the range of 0.01 to 10 µM (R^2^ = 0.9932–0.9998). The amount of DMBQ were calculated based on the standard curve and divided by the concentration factor to determine the DMBQ concentrations in the original samples. The lowest concentration of DMBQ in the standard curve divided by concentration factor was used as the limit of quantification (LOQ).

### Plasmid Construction

The PcUbi4 promoter (Kawalleck et al., 1993), mClover3 (Bajar et al., 2016), NOS terminator (Bandaranayake et al., 2010) were PCR amplified from their respective templates and cloned into the vector backbone pCAMBIA-0380 (GenBank: AF234290.1) between the restriction sites MauBI and SpeI using Gibson Assembly (NEB) to build pDS2, which allows visual selection of transgenic roots by mClover florescent protein. To build the parent expression vector pDS3 for oxidase genes, the CaMV 35S promoter and CaMV 35S terminator were PCR amplified from pHellsgate8 (Bandaranayake et al., 2010) and inserted into pDS2 backbone digested with EcoRI and AscI. Sequences of all vectors were confirmed by Sanger sequencing (DNA Sequencing Facility, UC Davis).

RNA was isolated from wild type *Arabidopsis* roots using the RNeasy Plant Mini Kit (Qiagen), treated with DNase I, and reverse transcribed to cDNA using High-Capacity cDNA Reverse Transcription Kit (Applied Biosystems). Seven different peroxidase genes (*AtPrx2*, AT1G05250; *AtPrx4*, AT1G14540; *AtPrx21*, AT2G37130; *AtPrx31*, AT3G28200; *AtPrx53*, AT5G06720; *AtPrx71*, AT5G64120; *AtPrx72*, AT5G66390) were amplified from the *Arabidopsis* cDNA. The primers used are listed in **Supplemental Table 1**. The laccase gene from white rot fungus *Trametes versicolor*, *TvLCC1* (GenBank ID: AY693776, Valderrama et al., 2005) was synthesized by Genscript (USA). Each amplified gene was inserted into pDS3 vector between PacI and SacI *via* Gibson Assembly (NEB). The vectors were verified by Sanger sequencing, transformed into *Rhizobium rhizogenes* stain MSU440 by electroporation (Sonti et al., 1995), and transformed into *Triphysaria* roots (Bandaranayake and Yoder, 2018).

### *Rhizobium rhizogenes*–Mediated Hairy Root Transformation in *Triphysaria*

Transgenic *Triphysaria* roots were obtained by *Rhizobium rhizogenes* transformation as described (Bandaranayake and Yoder, 2018) and maintained on square petri plates containing 0.25× Hoagland nutrient media, 0.75% (w/v) sucrose, 0.75% (w/v) Phytagel and 300 mg/L Timentin to control the growth of *Rhizobium rhizogenes*. For each construct transformed, number of independent plants with transgenic roots analyzed in this study are shown in parenthesis: 35S-AtPrx2 (19), 35S-AtPrx4 (25), 35S-AtPrx21 (19), 35S-AtPrx31 (16), 35S-AtPrx53 (23), 35S-AtPrx71 (27), 35S-AtPrx72 (16), 35S-TvLCC1 (22), empty vector control (19).

### RNA Isolation and Transcriptional Analysis

Transcript levels of transgenes in transgenic *Triphysaria* roots were determined by quantitative real-time PCR. Transgenic and control *Triphysaria* roots were harvested and immediately frozen in liquid nitrogen. The tissue was ground in liquid nitrogen and RNA was isolated using the RNeasy Plant Mini Kit (Qiagen). RNA was treated with DNase I prior to reverse transcription. One microgram or less RNA was converted to cDNA using High-Capacity cDNA Reverse Transcription Kit (Applied Biosystems). Each cDNA was diluted 50-fold and used for SYBR green-based real-time quantitative PCR assays in ABI 7300 quantitative PCR system (Applied Biosystems). At least three biological replicates were analyzed for each transgenic line.

Primer pairs for qPCR were validated by building standard curves with four 10-fold serial dilutions. Only primer pairs that showed over 90% amplification efficiency and generated a single peak in dissociation curve were selected (Sigma-Aldrich, 2012). Transcript levels were measured in three technical replicates for each transgenic root. Gene expression levels were calculated using the Delta-Delta C_T_ method relative to the geometric mean of two reference genes *TvQNA8* and *TvTUB1* (Vandesompele et al., 2002). The primers used are listed in **Supplemental Table 1**.

### Statistical Analysis

Statistical analysis was carried out in R. All the experiments with statistical analysis were completed with at least three biological replicates. Analysis of variance for experiments with more than two treatments were carried out by Tukey HSD (honestly significant difference) test.

## Results

### *Triphysaria* Rarely Parasitize Other *Triphysaria*

**Figure 1:**
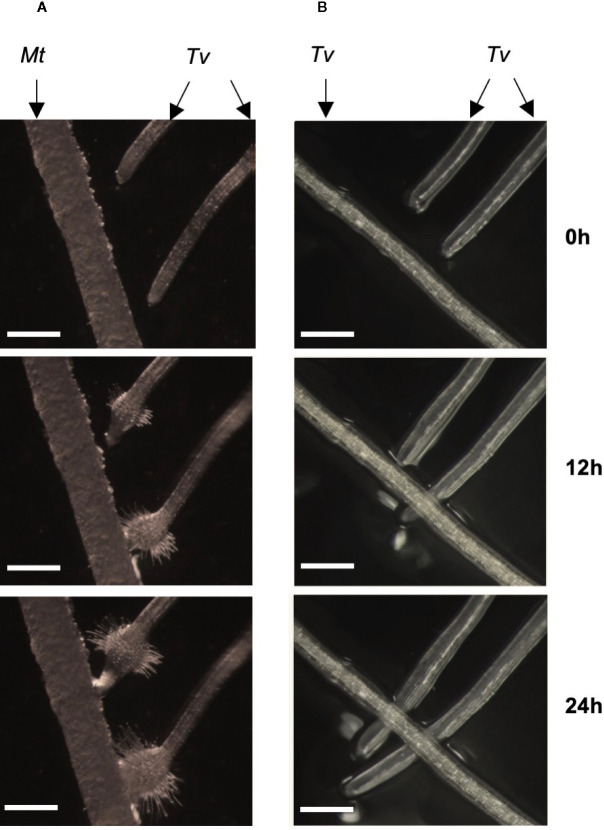
Kin recognition in *Triphysaria versicolor*. **(A)** Two *Triphysaria* seedlings were aligned to grow towards the root of *Medicago*. **(B)** *Triphysaria* seedlings were aligned to grow towards the root of another *Triphysaria*. *Tv*, *Triphysaria versicolor*. *Mt*, *Medicago truncatula*. Scale bar, 0.5 mm. Time-lapse videos are presented in **Supplemental Videos 1**, **2**.

*Triphysaria versicolor* is a generalist capable of parasitizing both monocot and dicot host species. When their roots contact those of the host plant *Medicago truncatula*, *Triphysaria* developed haustoria, globular root swellings with abundant localized hairs (**Figure 1A** and **Supplemental Video 1**). In certain cases, *Triphysaria* develop haustoria without touching host roots, indicating haustoria initiation is triggered by chemicals released by *Medicago* plants even in the absence of direct contact. Kin recognition was strikingly observed when aligning *Triphysaria* seedlings to grow towards the root of another *Triphysaria* (**Figure 1B** and the **Supplemental Video 2**). *Triphysaria* did not form haustoria on the other *Triphysaria* root but rather continued to grow past it.

### Haustorium-Inducing Activities Differ Between Host and Parasite Root Exudates

**Figure 2:**
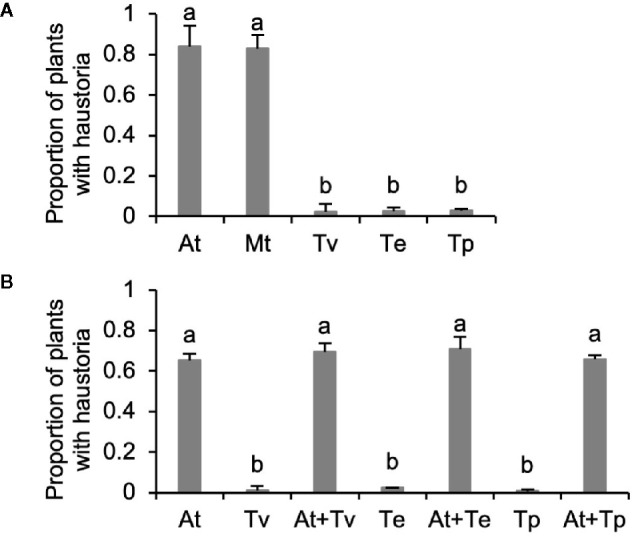
Haustorium-inducing activity of root exudates collected from *in vitro* root cultures. **(A)** All root exudates were normalized to 100 µM total phenolic contents. **(B)** Combination of *Arabidopsis* and *Triphysaria* root exudates that were all normalized to 30 µM total phenolic contents. *At*, *Arabidopsis thaliana*; *Mt*, *Medicago truncatula*; *Tv*, *Triphysaria versicolor*; *Tp*, *Triphysaria pusilla*; *Te*, *Triphysaria eriantha*. Error bars indicate standard deviation of three biological replicates. Significant (P < 0.05) differences between means by Tukey’s HSD test are indicated by different letters.

Because haustorium development is induced by chemical HIFs released in exudates of host roots, we hypothesize that kin recognition in *Triphysaria* may reflect differences in host and parasite root exudates. We grew root cultures of *Arabidopsis* and *Medicago* as representative host species and three *Triphysaria* species as parasites. The root culture growth media containing exudates were collected and concentrated with C18 solid phase extraction cartridges. Concentrated root exudates were normalized to the same total phenolic concentration (100 µM) using Folin-Denis assay. When 1 ml of the normalized exudates were applied to *Triphysaria* seedlings, the *Arabidopsis* and *Medicago* root exudates induced over 80% *Triphysaria* seedling to form haustoria, while the exudates of parasite origin induced very few (**Figure 2A**).

To determine if *Triphysaria* root exudates inhibited haustorium development, *Triphysaria* exudates were mixed with *Arabidopsis* root exudates before being applied to *Triphysaria* seedlings. We found that *Triphysaria* root exudates did not reduce the haustorium-inducing activity of *Arabidopsis* root exudates, indicating that *Triphysaria* exudates do not contain haustorium inhibitors (**Figure 2B**). These results show that kin recognition in *Triphysarria* is associated with *Triphysaria* root exudates missing active HIFs that can be recognized by other *Triphysaria*.

### Digestion With Phenol Oxidases Releases HIFs From *Triphysaria* Roots

**Figure 3:**
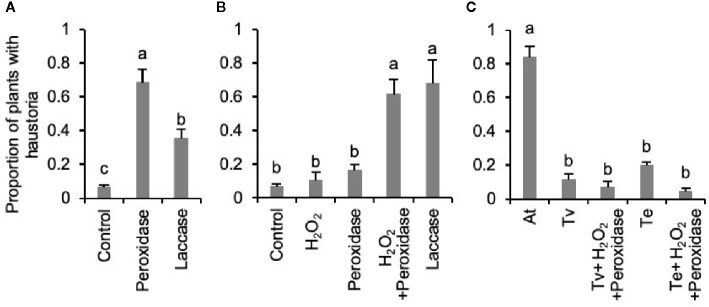
Release of haustorium inducing activities in *Triphysaria* roots by phenol oxidases. **(A)** Induction of haustorium development by directly applying horseradish peroxidase and fungal laccase (25 unit/ml). Control shows the no enzyme treatment. **(B)** Haustorium-inducing activity of enzymatic root extractions of intact *Triphysaria* roots by horseradish peroxidase or fungal laccase (25 unit/ml). **(C)** Haustorium-inducing activity of *Triphysaria* root exudates (100 µM) after oxidation by horseradish peroxidase (25 unit/ml). At, *Arabidopsis* root exudates at 100 µM total phenolic contents. Error bars indicate standard deviation of three biological replicates. Significant (P < 0.05) differences between means by Tukey’s HSD test are indicated by different letters.

Phenol oxidases have been proposed to be involved in both HIF synthesis and perception by parasitic plants (Kim et al., 1998; Keyes et al., 2000; Wada et al., 2019). To test if phenol oxidases are able to activate haustorium-inducing activity from *Triphysaria* roots, we applied two commercially available phenol oxidases, horseradish peroxidase and fungal laccase, directly to *Triphysaria* seedlings and found that they induced haustorium development (**Figure 3A**). To distinguish whether these enzymes directly induced haustoria or rather released HIFs which in turn activated haustoria development, we treated intact *Triphyaria* roots with peroxidase or laccase, filtered the enzymatic root extractions to remove the enzymes and assayed HIF activities by applying to another set of *Triphysaria* seedlings. **Figure 3B** showed that enzymatic extraction of intact *Triphysaria* roots with either horseradish peroxidase and H_2_O_2_ or fungal laccase released functional HIFs. To determine if root exudates themselves were activated by enzymatic oxidations, we incubated root culture exudates with peroxidase and analyzed their bioactivity on *Triphysaria* seedlings. **Figure 3C** showed that compounds in parasite root exudates were not oxidatively activated into HIFs, but rather substrates in *Triphysaria* roots were oxidized to produce active HIFs.

### *Triphysaria* Roots Transgenic for a Fungal Laccase Gene Show Enhanced Haustorium Development in the Presence of DMBQ

Because exogenous treatment of phenol oxidases to *Triphysaria* roots release haustorium-inducing activities, we hypothesize that the selective inactivity of phenol oxidases in *Triphysaria* may be responsible for kin recognition. To test this, we made transgenic *Triphysaria* roots containing constitutively expressed peroxidase genes from *Arabidopsis.* Class III peroxidase in *Arabidopsis* is a multi-gene family with 73 members and we selected seven secreted class III peroxidase genes to cover a wide range of phylogenetic divergency and substrate activities (Tognolli et al., 2002; Cosio and Dunand, 2009; Francoz et al., 2015). In addition, we selected *TvLCC1*, a laccase gene from the white rot fungus *Trametes versicolor* because this enzyme is among the best characterized fungal laccases and capable of oxidizing multiple substrates (Valderrama et al., 2005). In contrast to low number of isoforms and well-characterized biochemical activity of laccases in fungi, plant laccases consist multi-member gene families (Dwivedi et al., 2011). For examples, there are 17 laccase genes in *Arabidopsis thaliana* (Cai et al., 2006) and 93 laccase genes in soybean (Wang Q. et al., 2019). The specific function of each laccase gene in plants remain to be characterized, therefore we picked one fungal laccase gene to overexpress in *Triphysaria.* Each gene was driven by the CaMV 35S promoter and transformed into wild type *Triphysaria versicolor* by *Rhizobium rhizogenes* mediated root transformation (Bandaranayake and Yoder, 2018). Using this transformation method, *Triphysaria* plants became chimeric with the shoot remaining wild type while transgenic roots emerge from the shoot-root junction. Transgenic roots were selected using the mClover3, a Green Florescent Protein (GFP) reporter gene. Each chimeric transgenic plant formed multiple roots and those roots could be genetically identical or different from one another depending on when the transformation took place. Transgenic *Triphysaria* roots bearing empty vectors were used as controls.

**Figure 4:**
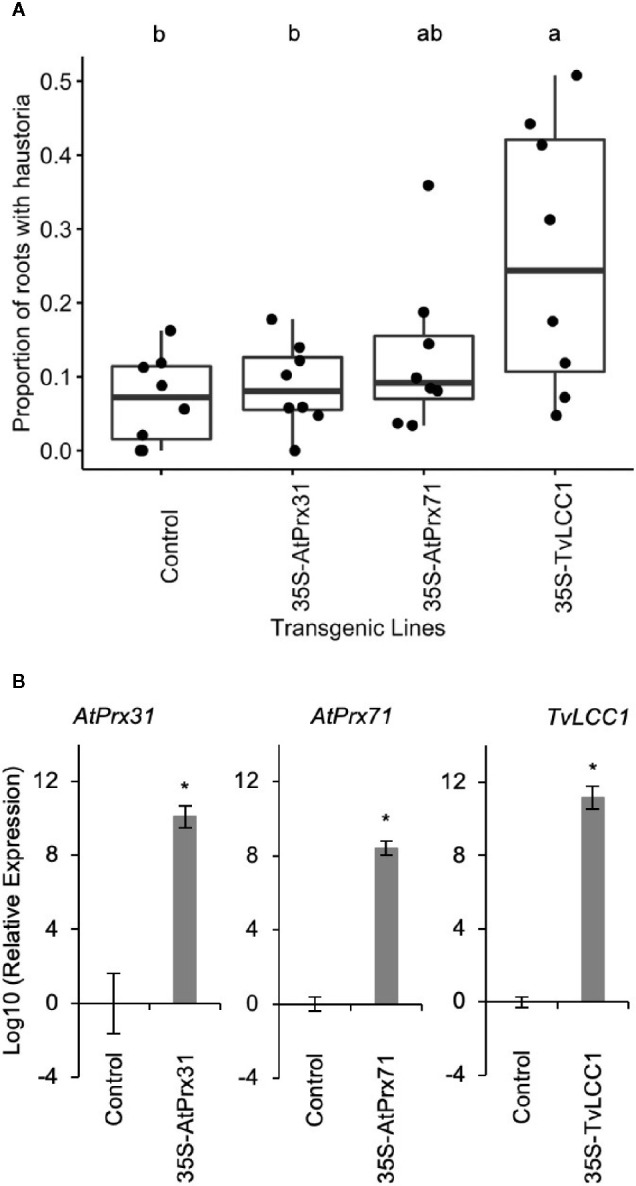
Phenotypic and transcript analysis on the transgenic roots. **(A)** Haustorium induction with 1 µM DMBQ. Transgenic roots generated on eight independent plants were assayed three times for each construct. Each dot in the graph indicates the average haustorium induction rates of the three replicates (number of roots on each transgenic plant = 16 ± 9). Significant (P < 0.05) differences between means by Tukey’s HSD test are indicated by different letters. **(B)** Transgene expression in transgenic *Triphysaria* roots by real-time quantitative PCR. Relative transcript levels were calculated based on geometric mean of two reference genes: *TvTUB1* and *TvQNA8*, then normalized to the expression levels in control lines and log10 transformed. Error bars indicate standard deviation of four to eight biological replicates, 35S-TvLCC1 (N=8), 35S-AtPrx31 (N=7), 35S-AtPrx71 (N=7), Control (N=4). *indicates significant difference (p-values < 0.05) between the control and transgenic lines by Student’s t-test.

Transgenic *Triphysaria* roots develop typical haustoria in morphology compared to wild-type seedlings when treated with HIFs (**Supplemental Figure 1**). We predicted that if ectopic expression of oxidases produced HIFs that haustoria may develop spontaneously in transgenic roots without the application of exogenous HIF. This was not the case. We carefully examined the transgenic roots under a dissecting scope without DMBQ induction and did not observe auto-haustoria formation in any of the peroxidase or laccase overexpressing lines. Therefore we assayed transgenic roots bearing each of the constructs by applying suboptimal amounts of DMBQ and monitoring transgenic roots for aberrant haustorium development. From the initial assay of nine constructs, three had significant increased sensitivity to DMBQ: *AtPrx31*, *AtPrx71* and *TvLCC1* (**Table 1**). Eight plants bearing transgenic roots from each of these three constructs that showed the highest haustorium formation rates were selected and assayed an additional three times. One transgenic construct, which overexpressed the fungal laccase gene *TvLCC1*, showed a statistically significant enhanced haustorium development rate in response to DMBQ compared to empty vector control (**Figure 4A**). The activity of this transgene was also apparent from its appearance: while most transgenic roots were phenotypically similar to control transgenic roots, transgenic roots overexpressing *TvLCC1* had abnormally brown primary roots (**Supplemental Figure 2**). Transgene expression levels were evaluated by real-time quantitative PCR and all transgenic roots showed high transgene expressions (**Figure 4B**). In summary, *in vivo* overexpression of fungal laccase gene *TvLCC1* in *Triphysaria* enhanced their haustorium formation ability in response to DMBQ.

**Table 1:** Initial screen of all transgenic roots treated with 1 µM DMBQ.

Transgene	Number of Plants	Total Roots	Roots with Haustoria	Proportion of Roots with Haustoria
Control	19	259	23	0.09
AtPrx2	19	139	7	0.05
AtPrx4	25	180	6	0.03
AtPrx21	19	135	7	0.05
AtPrx31	16	197	30	0.15*
AtPrx53	23	152	6	0.04
AtPrx71	27	201	40	0.20*
AtPrx72	16	101	5	0.05
TvLCC1	22	135	24	0.18*

Bold numbers with * indicate significant increase in haustorium formation rates (95% confidence interval = 0.05 – 0.14).

### *Triphysaria* Roots Do Not Release DMBQ in Exudates or by Phenol Oxidase Digestions

**Figure 5:**
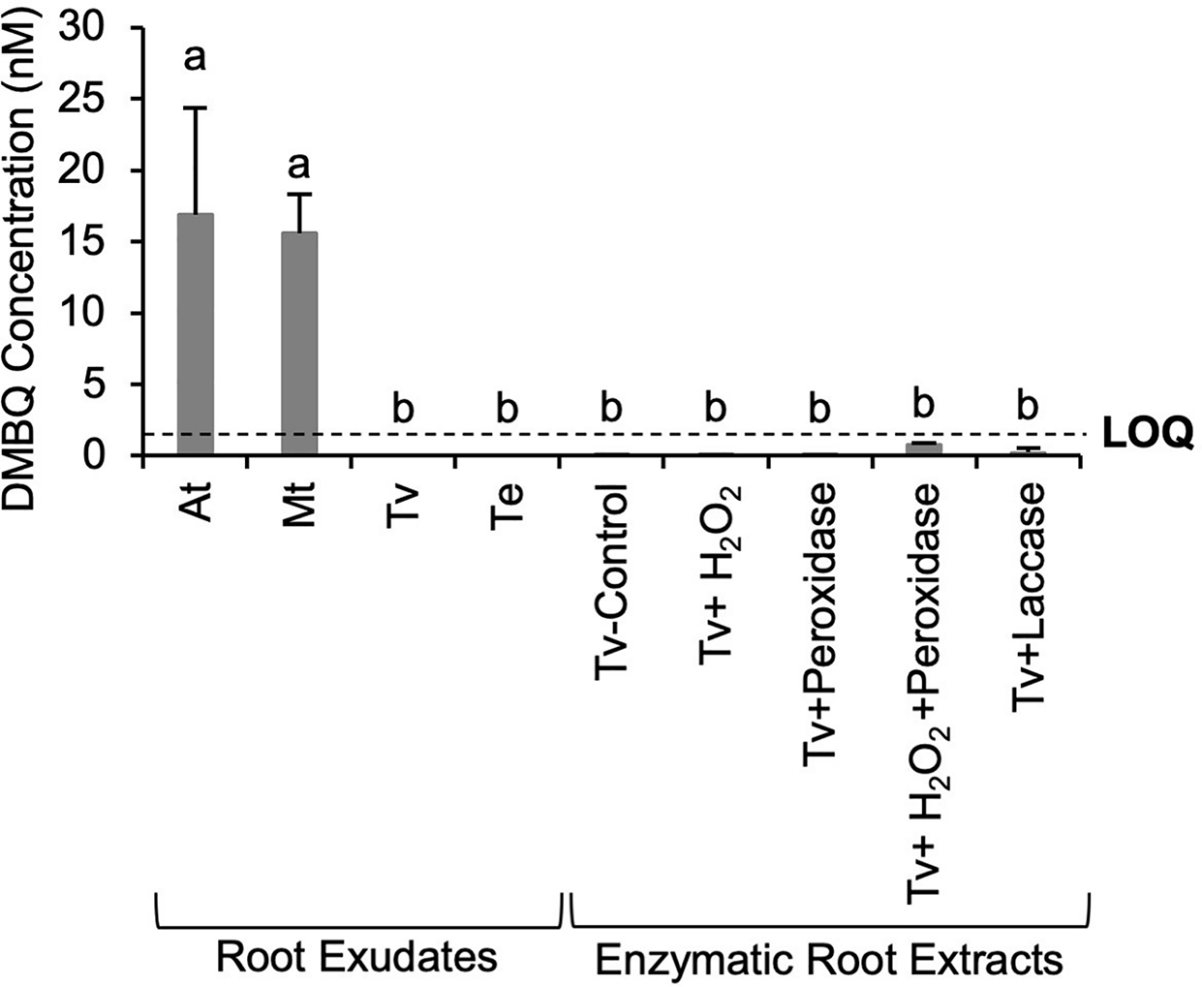
DMBQ quantification of root exudates and enzymatic extracts of *Triphysaria* roots by LC- triple quadrupole MS. Root exudates were concentrated from root culture media of different species and normalized to 100 µM total phenolic contents. Enzymatic root extractions of *Triphysaria* were collected by digesting intact *Triphysaria* roots with horseradish peroxidase (25 unit/ml), H_2_O_2_ or fungal laccase (25 unit/ml) in MES buffer (PH6). Tv-Control, *Triphysaria* roots incubated in MES buffer without enzymes. Error bars indicate standard deviation of three biological replicates. LOQ, limit of quantification, 1.5 nM. Significant (P < 0.05) differences between means by Tukey’s HSD test are indicated by different letters.

DMBQ is until now the only HIF identified from host roots and therefore was considered a likely candidate for what is contributing to the HIF activities of host root exudates and *Triphysaria* enzymatic root extracts by oxidases. The DMBQ concentrations in the root exudates and enzymatic extracts of *Triphysaria* roots were determined using LC-triple quadrupole MS. To validate our DMBQ quantification method, known amounts of DMBQ were spiked into the *Arabidopsis* root culture media before it was collected and concentrated. Root culture media with or without spiked DMBQ were processed in parallel and the DMBQ concentrations were calculated based on a standard curve using pure DMBQ (Millipore Sigma, USA). On average about 84% of the originally spiked DMBQ was detected (data not shown), indicating that the methodologies to extract and detect DMBQ from exudates were robust. This analysis revealed that phenol-normalized *Arabidopsis* and *Medicago* root exudates contained similar levels of DMBQ while DMBQ was not detected in parasite root exudates (**Figure 5**). Although enzymatic extracts of *Triphysaria* roots showed haustorium-inducing activities, DMBQ in those samples was either not detected or below the instrument limit of quantification (LOQ). These results suggested that the HIF activity differences between host and parasite roots exudates might be due to the difference in DMBQ concentrations and that the functional HIFs from enzymatic *Triphysaria* root extracts are not DMBQ.

## Discussion

*Triphysaria* is a generalist parasitic plant with a broad host range with one important exception; it rarely parasitizes its own roots or those of other *Triphysaria*. The avoidance of self or conspecific parasitism must have been an early event in parasite evolution to avoid unproductive parasitism of their own or sibling roots (Westwood et al., 2010). Our experiments show that root exudates from host plants induce haustoria when applied to *Triphysaria* roots but root exudates obtained from *Triphysaria* do not. Therefore *Triphysaria* roots are not releasing haustorium-inducing factors into the rhizosphere while many other plants are.

Plant root exudates are complex and evolutionarily optimized to coordinate signaling between many different organisms and plant roots. It seems more likely that plants release chemicals that trigger haustorium development into the rhizosphere not to attract parasites but rather parasitic plants use molecules produced by the plants for other purposes. For example, strigolactones play many functions in plant development, including coordination with mycorrhizae, but have also been coopted by some parasitic plants to trigger seed germination (Clarke et al., 2019). Because of the complexity of roles individual molecules may play in rhizosphere signaling, it remains difficult to predict the ecological costs associated with plant species that do or do not make specific signaling molecules.

A broad range of natural compounds have been identified as active HIFs through *in vitro* applications (Smith et al., 1996; Albrecht et al., 1999; Cui et al., 2018). While DMBQ is the most thoroughly studied HIF, several lines of evidence suggest that HIFs other than DMBQ are present in root exudates. A multi-generation selection from a field-collected self-pollinating *Triphysaria pusilla* population identified lineages that do not develop haustoria in response to DMBQ but do in response to host root exudates (Jamison and Yoder, 2001). In the current study, the concentration of DMBQ detected in *Arabidopsis* and *Medicago* root exudates accounted for only a small fraction of the HIF activity identified when exudates are assayed on *Triphysaria* seedlings. The concentration of DMBQ is positively correlated with its HIF activity and in the lab we typically induce haustoria in about 80% of *Triphysaria* seedlings with 10 to 30 µM DMBQ (Jamison and Yoder, 2001). *Arabidopsis* and *Medicago* root exudates also induced about 80% of *Triphysaria* to develop haustoria but the DMBQ concentrations in those samples was on average 16 nM, about three orders of magnitude lower than the level of pure DMBQ needed to achieve the same haustorium-inducing rate. These results suggest that *Arabidopsis* and *Medicago* root exudates include additional HIFs other than DMBQ or alternatively, exudates contain molecules which act synergistically with DMBQ to increase the efficiency of induction. Peroxidases and laccases oxidize phenolics to benoquinones, such as methyl-benzoquinone and methoxy-benzoquione, that have HIF activity (Caldwell and Steelink, 1969; Chang and Lynn, 1986; Jaufurally et al., 2016). These oxidative products may contribute to the unknown HIFs in *Triphysaria* enzymatic root extractions.

In a series of elegant experiments, Lynn and colleagues have shown that *Striga* seedlings are capable of oxidatively converting host cell wall phenolics into active HIFs (Lynn and Chang, 1990; Keyes et al., 2000; Keyes et al., 2007). They demonstrated that incubation of *Striga* *asiatica* seedlings with sorghum root surface material or syringic acid solution led to DMBQ formation and haustorium development. Their current model proposes that peroxidases in host cells are activated by hydrogen peroxide which is supplied by the *Striga* radicle. Host peroxidases then activate and release HIFs from their cell walls which then induces haustorium development in the same radicle donating the H_2_O_2_ substrate. In this way a *Striga* radicle oxidatively mines host cell walls for molecules to trigger haustorium development. We used this model as the basis to ask whether kin recognition in *Triphysaria* could be associated with masking the activity of HIF releasing peroxidases.

Horseradish peroxidase and fungal laccase are able to induce haustoria when directly applied to roots of *Triphysaria versicolor* or *Striga hermonthica* (Wada et al., 2019). We showed that peroxidase and laccase extractions of *Triphysaria* roots induced haustorium development when the enzymes were removed by filtration. This demonstrates that the products of enzymatic digestion of parasite roots contain HIFs which are necessary at the earliest stage in haustorium initiation. It is likely that peroxidases are also critical in later stages of haustorium development. Exposure to redox inhibitors prevent haustorium formation in *Striga* and *Triphysaria* even in the presence of DMBQ, suggesting that the activities of redox enzymes are indispensable for downstream processes after HIF perception (Wada et al., 2019; Wang Y. et al., 2019).

Our work is consistent with the lack of HIFs in *Triphysaria* root exudates but not host roots being caused by differential expression of oxidative enzymes in parasite versus host roots. Although oxidases such as peroxidase and laccase are abundantly present in plant roots, *Triphysaria* may restrict the activity of specific enzymes spatially to control the release of HIFs and prevent self-parasitism. Spatial separation of substrates and enzymes is a mechanism that regulates multiple biological processes. For examples, thioglucosidases (TGGs) are kept specifically in myrosin cells and only upon tissue damage TGGs can hydrolyze glucosinolates stored in vacuoles and produce components toxic to pathogens (Grubb and Abel, 2006; Wittstock and Burow, 2010). An alternative explanation is that *Triphysaria* cells walls do not contain the appropriate precursors for HIF synthesis. Host lignin composition affects haustorium initiation in parasitic plants *Striga hermonthica* and *Phtheirospermum japonicum* (Cui et al., 2018). It is possible that change in lignin or free phenolic composition in *Triphysaria* may result in production of inactive HIFs.

Our investigation of kin recognition in *Triphysaria* revealed the restricted release of HIFs in pbarasite but not host root exudates. HIFs were released from parasite cells by the exogenous applications or *in planta* overexpression of oxidases. We postulate that *Triphysaria* may spatially control the activity of phenol oxidases to prevent HIF production and the resulting self-parasitism. Further elucidation of the biochemical pathways associated with HIF production in host plants and clarification for their absence in parasites may lead to a mechanistic explanation of kin-recognition in parasitic plants and suggest a strategy to engineer crop plants and make them invisible to the parasitic weeds.

## Data Availability Statement

The original contributions presented in the study are included in the article/**Supplementary Materials**, further inquiries can be directed to the corresponding author/s.

## Author Contributions

YW and JY conceived the project. YW performed most of the experiments. MM and SL performed haustorium induction assays and root transformations. DS set up the imaging systems and contributed the videos of haustorium development. YW and JY wrote the article. YW, DS, and JY finalized the manuscript for submission. All authors contributed to the article and approved the submitted version.

## Funding

This work was supported by National Science Foundation grants DBI- 0701748 and IOS-1238057. YW and DS were supported by UC Davis Plant Sciences Department Graduate Student Research Fellowships and Henry A. Jastro Graduate Research Awards.

## Conflict of Interest

The authors declare that the research was conducted in the absence of any commercial or financial relationships that could be construed as a potential conflict of interest.

## Supplementary Material

Supplemental Table 1 Primers used in this study.

Supplemental Video 1 Time-lapse video of host recognition by *T. versicolor*. *T. versicolor* seedlings were aligned to grow towards the root of *Medicago truncatula*. Scale bar, 0.5 mm.

Supplemental Video 2 Time-lapse video of kin recognition by *T. versicolor. T. versicolor* seedlings were aligned to grow towards the root of another *T. versicolor.* Scale bar, 0.5 mm.

Supplemental Figure 1 Transgenic *Triphysaria* roots form typical haustoria in morphology. Both wild type seedlings and transgenic roots were induced with 30 μM DMBQ and fully developed haustoria formed 24 h after induction. Transgenic roots were transformed with the control vector overexpressing fluorescent protein mClover. Scale bar, 0.5 mm.

Supplemental Figure 2 Transgenic *Triphysaria* roots overexpressing fungal laccase *TvLCC1* showed browning root phenotype. Boxed areas are zoomed-in and showed in the bottom. Control transgenic roots were transformed with the empty vector pDS3, expressing only the fluorescent selectable marker mClover. Scale bar, 0.2 cm.
