# Supplementary material for: Kin Recognition in the Parasitic Plant Triphysaria versicolor Is Mediated Through Root Exudates
Source: Front Plant Sci. 2020 Oct 6;11:560682. doi: 10.3389/fpls.2020.560682 (PMC7573212; doi:10.3389/fpls.2020.560682)
Supplement: Supplemental Table 1 — Primers used in this study. [file Table_1.docx]

Supplemental Video 1. Time-lapse video of host recognition by *T. versicolor. T. versicolor* seedlings were aligned to grow towards the root of *Medicago truncatula*. Scale bar, 0.5 mm.

Supplemental Video 2. Time-lapse video of kin recognition by *T. versicolor*. *T.* versicolor seedlings were aligned to grow towards the root of another *T. versicolor*. Scale bar, 0.5 mm.

Supplemental Figure 1. Transgenic *Triphysaria* roots form typical haustoria in morphology. Both wild type seedlings and transgenic roots were induced with 30 µM DMBQ and fully developed haustoria formed 24 h after induction. Transgenic roots were transformed with the control vector overexpressing fluorescent protein mClover. Scale bar, 0.5 mm.

Supplemental Figure 2. Transgenic *Triphysaria* roots overexpressing fungal laccase *TvLCC1* showed browning root phenotype. Boxed areas are zoomed-in and showed in the bottom. Control transgenic roots were transformed with the empty vector pDS3, expressing only the fluorescent selectable marker mClover. Scale bar, 0.2 cm

Supplemental Table 1. Primers used in this study

| **Name** | **Sequence (5' to 3')** | **Purpose** |
| --- | --- | --- |
| AtPrx2-F | GAGAGGACACGGAGCTATGGCGATCAAGAACATTCTCG | Gene cloning |
| AtPrx2-R | GTGATTTTTGCGGACTTTAATTAATTAGTTAGGGAAGGCGCATCTCT |  |
| AtPrx4-F | GAGAGGACACGGAGCTATGGCGATCTTCAAGATTCTTGTATTG |  |
| AtPrx4-R | GTGATTTTTGCGGACTTTAATTAATTAATTAACGGCACTGCAGATTCTTCG |  |
| AtPrx21-F | GAGAGGACACGGAGCTATGGCCAATGCGAAGCCCTTTTG |  |
| AtPrx21-R | GTGATTTTTGCGGACTTTAATTAATTAGTTCACATAACGACAATCCTTCCTG |  |
| AtPrx31-F | GAGAGGACACGGAGCTATGGCTTCACTCAAATCTCTCTTCC |  |
| AtPrx31-R | GTGATTTTTGCGGACTTTAATTAATCAATTAATAGCATCGCACCTTCTTCG |  |
| AtPrx53-F | GAGAGGACACGGAGCTATGGCTGTAACAAATCTTCCTACTTGTG |  |
| AtPrx53-R | GTGATTTTTGCGGACTTTAATTAATCAACTTCCATTAACCTTCTTACAGTCTA |  |
| AtPrx71-F | GAGAGGACACGGAGCTATGGGTTTGGTTAGATCATTGTGCT |  |
| AtPrx71-R | GTGATTTTTGCGGACTTTAATTAATTAATTAACCGCAGAGCAAACCCTA |  |
| AtPrx72-F | GAGAGGACACGGAGCTATGGCCAAGTCATTGAACATCCTTA |  |
| AtPrx72-R | GTGATTTTTGCGGACTTTAATTAATTAATAAGCATGGTTAACCCTCCGA |  |
| TvLCC1-F | GAGAGGACACGGAGCTCATGGGTCTGCAGCGATTCAGCT |  |
| TvLCC1-R | GTGATTTTTGCGGACTTTAATTAATCACTGGTTAGCCTCGCTCAGC |  |
| TvTUB1-qPCR-F | CGGAAGGAAGCTGAGAACTGC | Quantitative real-time PCR |
| TvTUB1-qPCR-R | GAAGGGTTCCCATCCCAGAC |  |
| TvQNA8-qPCR-F | CAGCCCTACACAGCGGAAGA |  |
| TvQNA8-qPCR-R | TCAGACACGCCAATGAAGA |  |
| AtPrx31-qPCR-F | CACAGCTCAATCTCGCTTAACCAC |  |
| AtPrx31-qPCR-R | TCACGTTCTGCGGTGTTGAAG |  |
| AtPrx71-qPCR-F | GTCTCATCATCTCGGTTCATGGC |  |
| AtPrx71-qPCR-R | CGAACCGTCACAACCTTGGAC |  |
| TvLCC1-qPCR-F | CGTCGCTCGCTCTCTTGCAG |  |
| TvLCC1-qPCR-R | CGACGACGTTGAGCTGGAAGC |  |
