# Supplementary figures and images for: Kin Recognition in the Parasitic Plant Triphysaria versicolor Is Mediated Through Root Exudates
Source: Front Plant Sci. 2020 Oct 6;11:560682. doi: 10.3389/fpls.2020.560682 (PMC7573212; doi:10.3389/fpls.2020.560682)

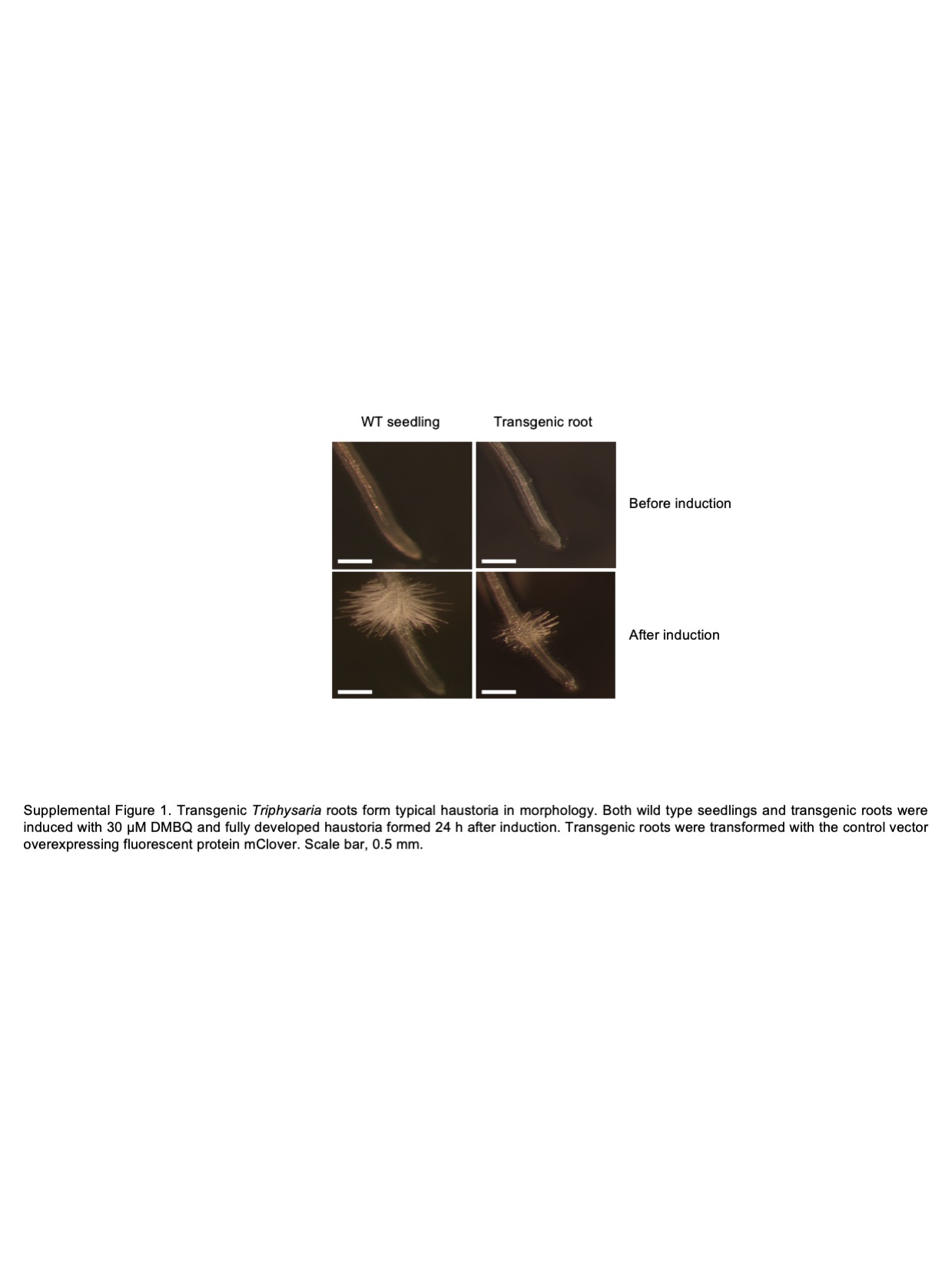

Supplement: Supplemental Figure 1 — Transgenic Triphysaria roots form typical haustoria in morphology. Both wild type seedlings and transgenic roots were induced with 30 μM DMBQ and fully developed haustoria formed 24 h after induction. Transgenic roots were transformed with the control vector overexpressing fluorescent protein mClover. Scale bar, 0.5 mm. [file Image_1.jpeg]

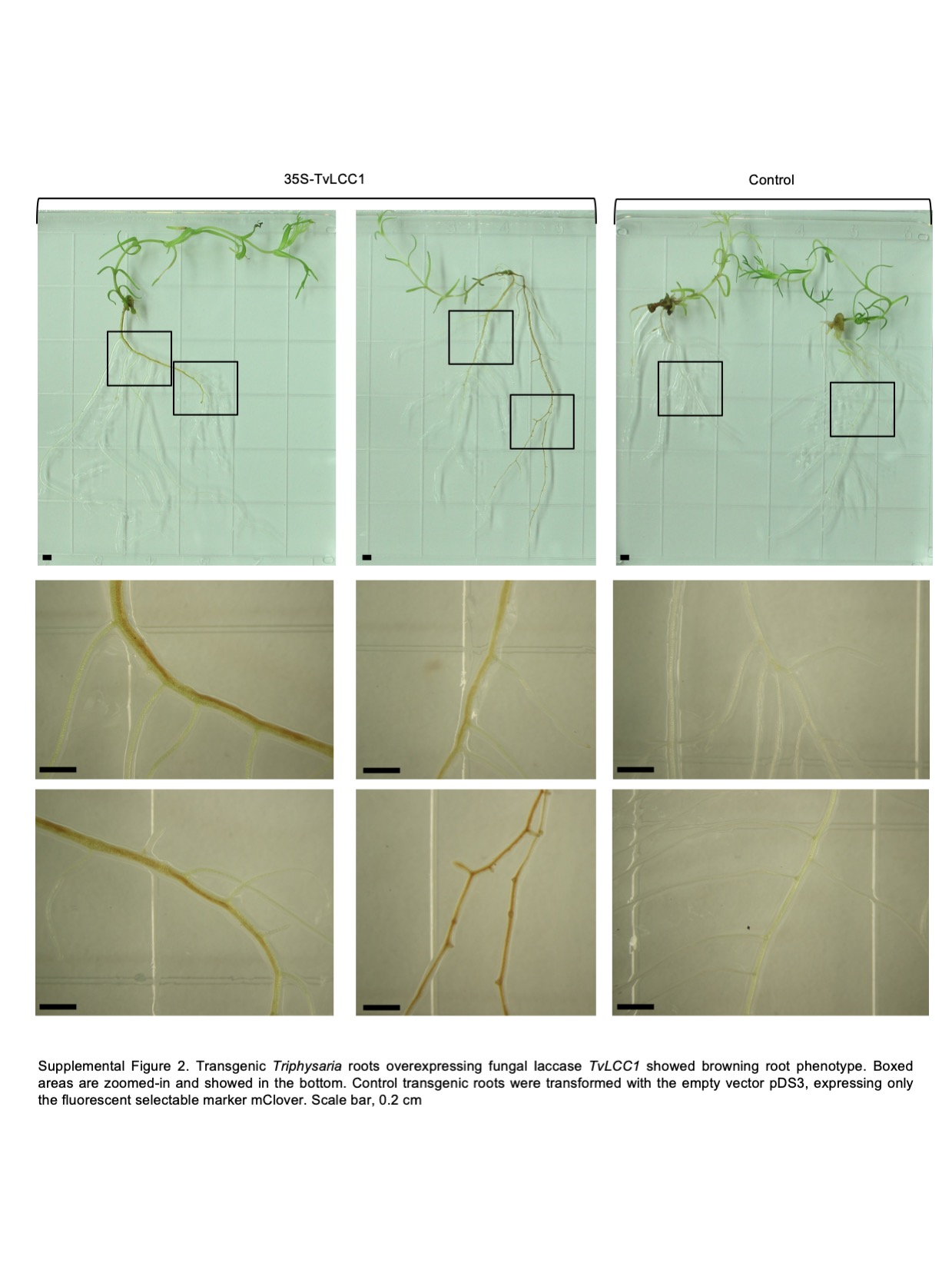

Supplement: Supplemental Figure 2 — Transgenic Triphysaria roots overexpressing fungal laccase TvLCC1 showed browning root phenotype. Boxed areas are zoomed-in and showed in the bottom. Control transgenic roots were transformed with the empty vector pDS3, expressing only the fluorescent selectable marker mClover. Scale bar, 0.2 cm. [file Image_2.jpeg]
